# Association of prenatal modifiable risk factors with attention-deficit hyperactivity disorder outcomes at age 10 and 15 in an extremely low gestational age cohort

**DOI:** 10.3389/fnhum.2022.911098

**Published:** 2022-10-20

**Authors:** David M. Cochran, Elizabeth T. Jensen, Jean A. Frazier, Isha Jalnapurkar, Sohye Kim, Kyle R. Roell, Robert M. Joseph, Stephen R. Hooper, Hudson P. Santos, Karl C. K. Kuban, Rebecca C. Fry, T. Michael O’Shea

**Affiliations:** ^1^Eunice Kennedy Shriver Center, UMass Chan Medical School, Worcester, MA, United States; ^2^Department of Epidemiology and Prevention, Wake Forest University School of Medicine, Winston-Salem, NC, United States; ^3^Department of Environmental Sciences and Engineering, Institute for Environmental Health Solutions, University of North Carolina School, Chapel Hill, NC, United States; ^4^Department of Anatomy and Neurobiology, Boston University School of Medicine, Boston, MA, United States; ^5^Department of Health Sciences, University of North Carolina School of Medicine, Chapel Hill, NC, United States; ^6^School of Nursing and Health Studies, University of Miami, Coral Gables, FL, United States; ^7^Division of Neurology (Pediatric Neurology), Department of Pediatrics, Boston Medical Center and Boston University, Boston, MA, United States; ^8^Department of Pediatrics, University of North Carolina School of Medicine, Chapel Hill, NC, United States

**Keywords:** attention-deficit hyperactivity disorder, maternal smoking, maternal obesity, maternal hypertension, preterm

## Abstract

**Background:**

The increased risk of developing attention-deficit hyperactivity disorder (ADHD) in extremely preterm infants is well-documented. Better understanding of perinatal risk factors, particularly those that are modifiable, can inform prevention efforts.

**Methods:**

We examined data from the Extremely Low Gestational Age Newborns (ELGAN) Study. Participants were screened for ADHD at age 10 with the Child Symptom Inventory-4 (*N* = 734) and assessed at age 15 with a structured diagnostic interview (MINI-KID) to evaluate for the diagnosis of ADHD (*N* = 575). We studied associations of pre-pregnancy maternal body mass index (BMI), pregestational and/or gestational diabetes, maternal smoking during pregnancy (MSDP), and hypertensive disorders of pregnancy (HDP) with 10-year and 15-year ADHD outcomes. Relative risks were calculated using Poisson regression models with robust error variance, adjusted for maternal age, maternal educational status, use of food stamps, public insurance status, marital status at birth, and family history of ADHD. We defined ADHD as a positive screen on the CSI-4 at age 10 and/or meeting DSM-5 criteria at age 15 on the MINI-KID. We evaluated the robustness of the associations to broadening or restricting the definition of ADHD. We limited the analysis to individuals with IQ ≥ 70 to decrease confounding by cognitive functioning. We evaluated interactions between maternal BMI and diabetes status. We assessed for mediation of risk increase by alterations in inflammatory or neurotrophic protein levels in the first week of life.

**Results:**

Elevated maternal BMI and maternal diabetes were each associated with a 55–65% increase in risk of ADHD, with evidence of both additive and multiplicative interactions between the two exposures. MSDP and HDP were not associated with the risk of ADHD outcomes. There was some evidence for association of ADHD outcomes with high levels of inflammatory proteins or moderate levels of neurotrophic proteins, but there was no evidence that these mediated the risk associated with maternal BMI or diabetes.

**Conclusion:**

Contrary to previous population-based studies, MSDP and HDP did not predict ADHD outcomes in this extremely preterm cohort, but elevated maternal pre-pregnancy BMI, maternal diabetes, and perinatal inflammatory markers were associated with increased risk of ADHD at age 10 and/or 15, with positive interaction between pre-pregnancy BMI and maternal diabetes.

## Introduction

Attention-deficit hyperactivity disorder (ADHD) is the most prevalent neurodevelopmental disorder in children, affecting approximately 5% of the general population, with at least another 5% having substantial difficulties with inattention, overactivity, and impulsivity but not meeting the threshold for diagnosis (Sayal et al., 2018). It is characterized by symptoms of inattention, hyperactivity/impulsivity, or both (American Psychiatric Association, 2013). Individuals with ADHD are more likely to fail to complete high school, suffer from depression, be arrested, engage in illicit drug use, and engage in violent behaviors (Erskine et al., 2016).

Children born very preterm are much more likely to be diagnosed with ADHD, with a recent follow-up at age 15 of the Extremely Low Gestational Age Newborn (ELGAN) cohort (Frazier et al., 2022) reporting that ADHD diagnoses were the most common psychiatric outcome (18% prevalence). It is unclear why children born preterm have an increased prevalence of ADHD. Several modifiable maternal risk factors during the perinatal period have been associated with the development of ADHD in offspring, including maternal smoking during pregnancy (MSDP) (Dong et al., 2018), maternal overweight status and/or obesity (Sanchez et al., 2018), pregestational and/or gestational diabetes (Xiang et al., 2018), and hypertensive disorders of pregnancy (HDP) (Maher et al., 2018). Other sibling cohort studies of MSDP have suggested that the observed relationship may be mostly due to familial or genetic confounding (Skoglund et al., 2014; Obel et al., 2016).

Our group has previously reported on the effects of these modifiable maternal risk factors on age 10 outcomes of the same cohort. Active smoking during pregnancy and maternal BMI ≥ 30 were identified as two of the risk factors associated with ADHD at age 10 (Leviton et al., 2018). However, the previous analysis used an empirical rather than theoretical method to determine potential confounders and neglected to control for family history of ADHD. Also, the analysis was limited to individuals with IQ ≥ 85. Our group has also previously reported that maternal overweight status or obesity was associated with increased ADHD behaviors at age 10, but the previously reported analysis did not control for family history of ADHD and did not assess the added effect of maternal diabetes (van der Burg et al., 2017). A previous report on the effect of prenatal tobacco exposure on neurological outcomes identified that active, but not passive, smoking was associated with an increased risk of epilepsy (Venkatesh et al., 2021). There was no effect on the risk of cerebral palsy or cognitive impairment. Evaluation of the antecedents of childhood obesity at age 10 in this same cohort identified pre-pregnancy maternal BMI ≥ 30 and gestational diabetes as risk factors (Wood et al., 2018).

We aimed to extend these previous studies to evaluate these risk factors in a longitudinal preterm cohort, considering ADHD outcomes at age 10 and 15 years. The current study builds on the previous work by: (1) extending the time period of assessment to incorporate age 10 and 15 data; (2) including the family history of ADHD as a potential confounder to control for potential genetic confounding; (3) including a broader IQ range than previous studies; and (4) incorporating ADHD medication status as an indicator of severity of ADHD to evaluate the robustness of the effects. Also, given the association between obesity and diabetes, we explored whether their effects on ADHD outcomes are independent or whether there may be additive or multiplicative effects of these interrelated risk factors. Therefore, we also assessed the interaction between elevated body mass index (BMI) and diabetes. Further, we examined the evidence for mediation of these effects by perinatal inflammation as indicated by elevations of inflammatory or neurotrophic proteins in the neonate. We hypothesized that: (1) MSDP, HDP, elevated maternal BMI, and/or maternal diabetes increase the risk of ADHD symptoms at age 10 and 15; (2) elevated maternal BMI and maternal diabetes demonstrate positive interaction in the increased risk of ADHD; and (3) perinatal inflammation is a common mediating pathway for these risk factors to contribute to increased ADHD risk.

## Materials and methods

### Participants

The ELGAN Study is a US multicenter prospective, observational study of the risk of structural and functional neurologic disorders in extremely preterm infants (O’Shea et al., 2009). The study enrolled 1,506 infants born before the 28th week of gestation from 2002 to 2004. At age 10 years, we recruited the 996 children for whom we had measurements of inflammation markers in blood specimens collected during the first postnatal month. Of the 996 eligible children, 889 (89%) were enrolled (Joseph et al., 2017). Of this number, we limited our current analysis to those with an IQ ≥ 70, and a completed Child Symptom Inventory, Parent Checklist (CSI-4), at age 10 years. At age 15 years, we attempted to recruit all 1,198 surviving members of the cohort. A total of 700 adolescents (58% of surviving members) were evaluated at age 15, and 670 of them (96%) were interviewed using the Mini-International Neuropsychiatric Interview for Children and Adolescents (MINI-KID). The 754 participants with either a CSI-4 from age 10 or a MINI-KID from age 15 available and with IQ ≥ 70 constituted the sample for this report. Enrollment and consent procedures for this follow-up study were approved by the institutional review boards of all participating institutions. The caregivers provided their written informed consent to participate in this study, and the participants provided their assent if developmentally able to do so.

### Demographic and pregnancy variables

After delivery, a trained research nurse interviewed each mother in her native language using a structured data collection form and obtained information about characteristics of the mother and pregnancy, as well as exposures during pregnancy.

### Infant characteristics

Neonatal data were collected from the newborn’s medical record. The gestational age estimates were based on a hierarchy of the quality of available information as reported previously (van der Burg et al., 2017). The birth weight *z*-score represents the number of standard deviations the infant’s birth weight was above or below the median weight of infants at the same gestational age in referent samples not delivered for preeclampsia or fetal indications (Yudkin et al., 1987; Leviton et al., 1999).

#### Evaluation of neonatal systemic inflammation

As described in more detail elsewhere (Fichorova et al., 2011; Leviton et al., 2011; Dammann et al., 2016), drops of blood were collected on filter paper at times when study participants were having blood collected to guide clinical care. When available, during the first 2 postnatal weeks, 3 specimens were collected from each participant at the following times: day-1 (range, 1–3 days), day-7 (range, 5–8 days), and day-14 (range, 12–15 days). Blood drops were collected on filter paper (Schleicher & Schuell 903, GE Healthcare, Chicago, IL, United States) and stored at −70°C in sealed bags with desiccant until processing. Blood specimens were assayed for inflammation-related proteins in the Laboratory of Genital Tract Biology, Brigham and Women’s Hospital. Detailed description of protocols for protein elution, procedures for measuring concentrations, as well as absolute values and ranges of proteins quantified have been described elsewhere (Leviton et al., 2012; Fichorova et al., 2015; Allred et al., 2017; Kuban et al., 2017, 2019). As described previously (Kuban et al., 2017, 2019), of the 27 inflammation-related proteins that were assayed, six have been associated most consistently with structural and/or functional neurologic outcomes in previous ELGAN studies and were thus the focus of this study. These six proteins are: interleukin-6 (IL-6), tumor necrosis factor-alpha (TNF-α), intercellular adhesion molecule-1 (ICAM-1), interleukin-8 (IL-8), serum amyloid A (SAA), and C-reactive protein (CRP). For each of these six proteins, we dichotomized protein levels at the 75th percentile as the criterion for a protein elevation. Infants with a protein elevation on at least 2 days during the first two postnatal weeks were classified as having an intermittent or sustained elevation of that protein (Leviton et al., 2012). Children were classified as having low, moderate, or high levels of inflammatory proteins if they had sustained elevation of 0, 1–2, or ≥3 of these six proteins (Kuban et al., 2019).

#### Evaluation of neurotrophic proteins

As reported previously (Kuban et al., 2019), we also categorized children based on sustained elevation of the following 12 neurotrophic proteins: IL-6R, regulated upon activation, and normal T-cell expressed, and (presumably) secreted (RANTES), brain-derived neurotrophic factor (BDNF), basic fibroblast growth factor, insulin-like growth factor-1, vascular endothelial growth factor, vascular endothelial growth factor receptor-1, vascular endothelial growth factor receptor-2, placental growth factor, angiopoietin 1 (ANG-1), ANG-2, and thyroid-stimulating hormone. We summed the number of these 12 proteins with sustained elevation and categorized children as having low (0–1), moderate (2–3), or high (≥4) levels of elevated neurotrophic proteins.

### Full-scale intelligence quotient

Cognitive function was assessed at age 10 years with the Differential Ability Scales-II (Elliott, 1990) Verbal and Non-verbal Reasoning subscales, which were averaged to generate an estimate of full-scale IQ. At 15 years, full-scale IQ was measured with the Wechsler Abbreviated Scale of Intelligence-II (WASI-II) two-subtest form (Wechsler, 2011), which estimates full-scale IQ from the Vocabulary and Matrix Reasoning subtests of the WASI-II. The sample in the analysis excluded those with full-scale IQ < 70 at age 10 or age 15, to minimize confounding of ADHD symptoms by factors associated with intellectual disability (Sawhney et al., 2021). As a sensitivity analysis to evaluate the robustness of our results, we repeated the main exposure-outcome analyses with all participants regardless of IQ, including IQ as a covariate in the regression models.

### Exposures

#### Maternal body mass index

Each mother was asked to provide her height and pre-pregnancy weight shortly before, or shortly after, delivery when they were interviewed, usually by a research nurse. These data were used to calculate her BMI. The United States government classifies BMIs as follows: <18.5 kg/m^2^ is underweight, 18.5–24.9 kg/m^2^ is normal, 25.0–29.9 kg/m^2^ is overweight, 30.0–34.9 kg/m^2^ is obese, 35.0–39.9 kg/m^2^ is very obese, and ≥40 kg/m^2^ is extreme obesity (NIH). We collapsed these BMI groups into <25 (reference), 25–29.9 (overweight), and ≥30 kg/m^2^ (obese).

#### Maternal smoking during pregnancy

Prenatal maternal passive and active tobacco smoke exposure was ascertained by maternal interview at the time of participant enrollment (Venkatesh et al., 2021). In this analysis, active maternal smoke exposure during pregnancy was assessed. As a sensitivity analysis, we also assessed the effects of passive maternal smoke exposure during pregnancy and any combination of active or passive maternal smoke exposure during pregnancy.

#### Maternal hypertensive disorders of pregnancy

Maternal hypertensive disorders diagnosed before pregnancy was ascertained by maternal interview around the time of participant enrollment. Maternal hypertensive disorders during pregnancy, including pregnancy-induced hypertension, preeclampsia, eclampsia, and HELLP (Hemolysis, Elevated Liver enzymes, and Low Platelets) syndrome, were ascertained by study coordinators’ reviews of maternal obstetric records.

#### Maternal diabetes

Pre-pregnancy maternal diabetes and gestational diabetes were ascertained by maternal interview around the time of participant enrollment. The interview did not distinguish between type 1 and type 2 pre-pregnancy diabetes. We defined maternal diabetes as having either pre-pregnancy maternal diabetes and/or gestational diabetes. We did not obtain data on diabetes management during pregnancy.

### Outcomes

#### Attention-deficit hyperactivity disorder outcome at age 10

At the 10-year assessment, the parent or caregiver completed questionnaires regarding the child’s medical and neurological status and behavior, including the Child Symptom Inventory-4, Parent Checklist (CSI-4) (Gadow and Sprafkin, 2002). The parent form of the CSI-4 contains 97 items that screen for 15 emotional and behavioral disorders, including 18 ADHD symptoms (nine for the inattentive domain and nine for the hyperactive/impulsive domain) that are each rated on a Likert scale from 0 (never) to 3 (very often). ADHD classifications were assigned based on the cut-off scores provided in the CSI-4 manual (Gadow and Sprafkin, 2002). We limited our age 10 analysis to only the parent-report CSI-4, because (i) the teacher report was only available for a smaller subset of participants (Leviton et al., 2018), (ii) previous analysis of the age 10 data indicated that agreement between multiple reporters did not provide more information about functional outcomes than that of a single reporter (Leviton et al., 2017), and (iii) this more closely aligned with available data from age 15 which primarily consisted of self-report during MINI-KID administration.

#### Attention-deficit hyperactivity disorder outcome at age 15

The MINI-KID is a structured clinical diagnostic interview designed to assess the presence of current DSM-5 and ICD-10 psychiatric disorders in youths aged 6–17 years (Sheehan et al., 2010). The interview is typically administered to the adolescent alone but can be administered together with the parent who accompanies the adolescent. For the participants with a MINI-KID assessment (*N* = 575), the majority (*N* = 564) of adolescents were interviewed alone, with a small subset either supplemented with parent report (*N* = 9) or diagnosed by parent interview alone (*N* = 2). The MINI-KID follows the structure and format of the adult version of the interview, which has been validated against the Structured Clinical Interview for DSM-III-R and against the World Health Organization–designed Composite International Diagnostic Interview. The MINI-KID has diagnostic sections/modules and is administered using branching tree logic (e.g., 2–4 screening questions per disorder, with additional questions asked only if the screen questions are endorsed). The instrument screens for 24 DSM-5 and ICD-10 psychiatric disorders and for suicidality. The MINI-KID has substantial to excellent concordance with the gold standard Schedule for Affective Disorders and Schizophrenia for School-Age Children–Present and Lifetime Version. The MINI-KID has been updated to version 7.0.2 to map onto DSM-5 diagnostic criteria. For this report, we used a current MINI-KID diagnosis of ADHD (any subtype) consistent with DSM-5 duration of past 6 months. Those who met criteria for current DSM-5 diagnosis of ADHD were included in the outcome group.

#### Medication-related outcomes at age 10 or 15 for sensitivity analyses

In order to test for sensitivity of our results to more stringent or inclusive criteria, we also used data from parent interviews at age 10 and age 15 about current or past medications being taken for ADHD treatment. We identified participants as being on medication for ADHD if the parent identified any current or past use of any ADHD medication, and the primary author confirmed that the medication was either a stimulant, alpha-2-agonist, or atomoxetine. For a more inclusive definition of the ADHD outcome, we included children who were not identified as positive for ADHD by CSI-4 at age 10 or by MINI-KID at age 15, but who had taken medication for ADHD. Presumably the children on medications, but without meeting outcome criteria, had symptoms of ADHD in the past that were either resolved or were well-managed by medication. For more stringent criteria for ADHD outcome, we included only those children who were identified as positive for ADHD by CSI-4 at age 10 or by MINI-KID at age 15, and also reported current or past use of medication for ADHD at age 10 or 15. Presumably these children had symptoms of ADHD that were severe enough to continue to meet criteria even with medication treatment.

### Data analysis

We evaluated the relationship between the various definitions of ADHD and maternal exposures, previously defined. To estimate the relative risk of ADHD with 95% confidence intervals (CI), Poisson regression models were fit. A robust error variance procedure was used to relax variance assumptions (Zou, 2004). All models were additionally adjusted for potential confounding by socioeconomic factors (maternal age, maternal education status, maternal food and nutritional services assistance, maternal public insurance status, maternal marital status), as well as potential genetic confounding (familial history of ADHD). Adjustment variables were determined using directed acyclic graphs, DAGs, to determine a minimally sufficient set of adjustment variables for inclusion in the models. Models assessing maternal smoke exposure and maternal BMI as main predictors were co-adjusted, as it was assumed that smoking may confound the BMI-ADHD relationship and vice versa. Models assessing maternal diabetes or maternal hypertension as the main predictor were additionally adjusted for potential confounding by maternal smoke exposure and maternal BMI. As an added test of robustness, we also evaluated the relationship between the exposures and ADHD outcomes using only age 10 data and only age 15 data. To evaluate the robustness of effects to IQ, the models were repeated in a supplementary analysis using all participants regardless of IQ and adjusting for IQ as a confounding covariate.

To investigate modification of the effect of maternal BMI on ADHD by maternal diabetes, interaction models were used. Similar to the previous analyses, Poisson regression models with a robust error variance were fit to estimate the relative risk of ADHD. To assess this interaction, overweight or obese, as previously defined, were combined into one overweight or obese maternal BMI category. An interaction term between maternal diabetes and the overweight/obese variable was included in the model adjusting for maternal age, maternal education status, maternal food and nutritional services assistance, maternal public insurance status, maternal marital status, and any familial history of ADHD. Assessment of additive and multiplicative interaction was performed using standard calculations for relative excess risk due to interaction, proportion of risk attributable to interaction, synergy index, and ratio of relative risks (Knol et al., 2007; Knol and VanderWeele, 2012).

To test for mediation effects of inflammatory or neurotrophic proteins, we followed a traditional 3-step regression framework (Baron and Kenny, 1986). First, analysis of the total effect of the independent variables (elevated maternal BMI/diabetes) on the dependent variables (ADHD outcomes) was conducted as described in the preceding paragraphs. Second, we assessed the effect of the mediator (inflammatory or neurotrophic proteins) on the dependent variables and the effect of the independent variables on the mediator, using Poisson regression models with robust error variance. The effect of inflammatory or neurotrophic proteins on ADHD outcomes was adjusted for potential confounding by socioeconomic factors (maternal age, maternal education status, maternal food and nutritional services assistance, maternal public insurance status, maternal marital status), and family history of ADHD. The effect of maternal BMI or diabetes on inflammatory or neurotrophic proteins was adjusted for potential confounding by socioeconomic factors as above, HDP, and MSDP. If the independent variables (BMI/diabetes) are significantly associated with the mediator (inflammatory or neurotrophic proteins) and the dependent variables (ADHD outcomes), and the mediator is significantly associated with the outcomes, then the direct and indirect effects can be calculated by evaluating the independent variable and mediator effects simultaneously on the dependent variable.

All analyses were conducted in R version 4.1.2 (R Core Team, 2021). The R package interactionR (Babatunde, 2021) was used to calculate relative risks and confidence intervals as well as interaction estimates and their respective *p*-values.

## Results

### Sample characteristics

**FIGURE 1:**
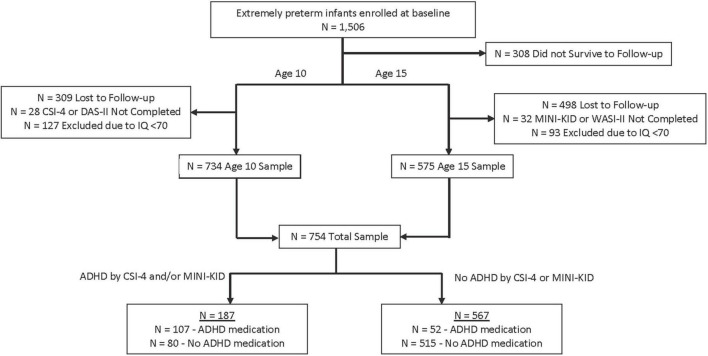
Flowchart for sample description. The total sample is not a sum of age 10 and age 15 numbers due to overlap of those assessed at both timepoints (*N* = 555). CSI-4, Child Symptom Inventory-4; DAS-II, Differential Ability Scale-II; MINI-KID, Mini International Neuropsychiatric Interview for Children and Adolescents; WASI-II, Wechsler Abbreviated Scale of Intelligence-II.

The sample flowchart for sample selection is shown in Figure 1. Demographic characteristics of the sample collected at birth are presented in Table 1, comparing our analysis sample to those who survived to the last study contact but were lost to follow-up before age 10 or who were missing ADHD outcomes at age 10 or 15. A higher proportion of participants in our sample was older, married, White, non-Hispanic, had completed high school, was not receiving public health insurance, and had pregestational or gestational diabetes.

**TABLE 1:** Description of sample (*N* = 754).

		In sample (%)	Not in sample (%)
**Maternal characteristics**			
Racial identity	White	502 (66.6%)	150 (50.0%)
	Black	171 (22.7%)	91 (30.3%)
	Other	77 (10.2%)	52 (17.3%)
Hispanic	Yes	71 (9.42%)	57 (19.0%)
Age at delivery, years	<21	95 (12.6%)	53 (17.7%)
	21–35	496 (65.8%)	203 (67.7%)
	>35	163 (21.6%)	44 (14.7%)
Education, years	≤12	275 (36.5%)	151 (50.3%)
	>12, <16	169 (22.4%)	64 (21.3%)
	≥16	287 (38.1%)	64 (21.3%)
Single marital status	Yes	282 (37.4%)	157 (52.3%)
Public insurance	Yes	236 (31.3%)	151 (50.3%)
Smoking during pregnancy	Yes	100 (13.3%)	38 (12.7%)
Passive smoking	Yes	169 (22.4%)	82 (27.3%)
Pre-pregnancy BMI	<25	435(57.7%)	170 (56.7%)
	≥25, <30	140 (18.6%)	56 (18.7%)
	≥30	151 (20.0%)	55 (18.3%)
Hypertension before or during pregnancy	Yes	133 (17.6%)	54 (18.0%)
Hypertension during pregnancy only	Yes	110 (14.6%)	45 (15.0%)
Pregestational or gestational diabetes	Yes	56 (7.43%)	11 (3.67%)
**Perinatal characteristics**			
Cesarean delivery	Yes	505 (67.0%)	201 (67.0%)
**Newborn characteristics**			
Sex	Male	366 (48.5%)	159 (53.0%)
Singleton	Yes	484 (64.2%)	218 (72.7%)
Gestational age, weeks	23–24	136 (18.0%)	60 (20.0%)
	25–26	344 (45.6%)	145 (48.3%)
	27	274 (36.3%)	95 (31.7%)
Birth weight, grams	≤750	248 (32.9%)	106 (35.3%)
	751–1000	346 (45.9%)	129 (43.0%)
	>1000	160 (21.2%)	65 (21.7%)
Birth weight *Z*-score	<−2	42 (5.57%)	9 (3.00%)
	≥−2, <−1	92 (12.2%)	36 (12.0%)
	≥−1	620 (82.2%)	255 (85.0%)
**Postnatal characteristics**			
Mycoplasma	Yes	66 (8.75%)	30 (10.0%)
Mech ventilation, Day 7	Yes	437 (58.0%)	155 (51.7%)
Retinopathy of prematurity	Yes	488 (64.7%)	187 (62.3%)
Necrotizing enterocolitis, bell stage III or isolated perforation	Yes	54 (7.16%)	20 (6.67%)
Antibiotics, first 4 weeks	Week 1	736 (97.6%)	299 (99.7%)
	Weeks 2–4	564 (74.8%)	230 (76.7%)
Ventriculomegaly	Yes	61 (8.09%)	24 (8.00%)
**Proteins**			
Inflammatory	0	445 (59.0%)	173 (57.7%)
	1–2	210 (27.9%)	97 (32.3%)
	3+	99 (13.1%)	30 (10.0%)
Neurotrophic	0–1	405 (53.7%)	154 (51.3%)
	2–3	186 (24.7%)	83 (27.7%)
	4+	163 (21.6%)	63 (21.0%)

Included in the table are the 1,054 individuals with IQ ≥ 70 who survived until age 10 or the last contact with the study team. Those included in the group not in the sample (*N* = 300) for this analysis are those who were either lost to follow-up or did not have IQ data or ADHD outcomes.

### Characterization of attention-deficit hyperactivity disorder outcomes in sample

Table 2 shows the characteristics of the ADHD outcomes and medication status for the participants included in this analysis. Of the entire sample, 74% were assessed at both age 10 and 15, 24% at age 10 alone, and 2.6% at age 15 alone. The table demonstrates the potential for excluding individuals with significant ADHD symptoms by history if only a single time point or assessment were used to define the outcome. Of those who were assessed at both time points and screened positive for ADHD symptoms at age 10 (*N* = 101), 62% were not diagnosed with ADHD by interview at age 15. Likewise, of those assessed at both time points and diagnosed with ADHD at age 15 (*N* = 87), 56% had not screened positive for symptoms at age 10. Nine percent (*N* = 52) of the participants who did not meet criteria for ADHD at either age 10 or 15 (*N* = 567) were either currently or previously treated with medications for ADHD, and 43% of those who met criteria for ADHD at either age 10 or 15 had never been treated with medications for ADHD.

**TABLE 2:** Characterization of ADHD outcomes and medication status in sample used for analysis.

	ADHD-, 15 years			ADHD+, 15 years			Missing 15 years data	
	ADHD-, 10 years	ADHD+, 10 years	Missing 10 years data	ADHD-, 10 years	ADHD+, 10 years	Missing 10 years data	ADHD-, 10 years	ADHD+, 10 years
No ADHD medications, *N*	366 (48.5%)	30 (4.0%)	16 (2.1%)	18 (2.4%)	8 (1.1%)	1 (1.3%)	133 (17.6%)	23 (3.0%)
History of ADHD medications, *N*	39 (5.2%)	33 (4.4%)	2 (0.3%)	31 (4.1%)	30 (4.0%)	1 (1.3%)	11 (1.4%)	12 (1.6%)

Percents are percents of total sample (*N* = 754).

### Effect of exposures on attention-deficit hyperactivity disorder outcomes

Table 3 demonstrates the crude and adjusted relative risks for ADHD outcomes for exposures to MSDP, elevated maternal BMI, pregestational and/or gestational diabetes, and maternal HDP. MSDP and HDP had no significant impact on the risk of ADHD outcomes at age 10 or 15. Sensitivity analyses for the effect of passive smoking only or any smoking (active or passive) resulted in similar non-significant relative risks ranging from 0.89 to 1.08 (data not shown). Overweight status increased the risk of ADHD outcome by 55% (95% CI: 11%–117%), and maternal obesity increased the risk by 65% (95% CI: 19%–128%). Finally, maternal diabetes increased the risk of ADHD by 56% (95% CI: 5%–131%). Using more stringent or more inclusive criteria for ADHD category by including ADHD medication status did not change the direction of the relationships. However, the magnitude of the increase in risk was higher when more stringent criteria were used, and lower with more inclusive criteria. Similar relationships were seen using only ADHD outcomes at age 10 (*N* = 734) and using only ADHD outcomes at age 15 (*N* = 575), as shown in the last two columns of Table 3. When the analysis was repeated with all participants regardless of IQ, using IQ as a covariate, the significant increased relative risk of ADHD with maternal elevated BMI and maternal diabetes remained (see Supplementary Table 1; *N* = 902).

**TABLE 3:** Crude and adjusted analyses for ADHD outcomes.

	ADHD 10 or 15	ADHD 10 or 15 AND medication	ADHD 10 or 15 OR medication	ADHD 10	ADHD 15
**Crude analyses**					
MSDP	1.28 (0.92–1.76)	1.13 (0.7–1.85)	1.22 (0.92–1.6)	1.55 (1.07–2.24)	1.39 (0.85–2.29)
Maternal PP BMI 25–30	1.45 (1.06–1.98)	1.62 (1.05–2.49)	1.28 (0.98–1.68)	1.62 (1.1–2.39)	1.7 (1.07–2.72)
Maternal PP BMI > 30	1.63 (1.22–2.18)	1.73 (1.14–2.61)	1.5 (1.18–1.91)	1.93 (1.36–2.74)	1.71 (1.09–2.68)
Maternal diabetes	1.46 (1–2.13)	1.69 (1.02–2.83)	1.3 (0.93–1.82)	1.7 (1.11–2.61)	1.48 (0.8–2.72)
Hypertension before or during pregnancy	1.05 (0.77–1.45)	1.26 (0.83–1.92)	1.1 (0.84–1.42)	1.05 (0.72–1.54)	1.05 (0.65–1.71)
Hypertension during pregnancy only	1.12 (0.8–1.56)	1.35 (0.87–2.09)	1.18 (0.9–1.55)	1.04 (0.68–1.57)	1.19 (0.73–1.95)
**Adjusted analyses** *					
MSDP	0.88 (0.59–1.31)	0.7 (0.41–1.22)	0.95 (0.67–1.34)	1.02 (0.64–1.63)	1.09 (0.65–1.85)
Maternal PP BMI 25–30	1.55 (1.11–2.17)	1.73 (1.12–2.68)	1.39 (1.04–1.86)	1.88 (1.21–2.92)	1.72 (1.08–2.74)
Maternal PP BMI > 30	1.65 (1.19–2.28)	1.88 (1.23–2.87)	1.64 (1.25–2.13)	2.13 (1.42–3.2)	1.84 (1.14–2.98)
Maternal diabetes	1.56 (1.05–2.31)	1.73 (1.01–2.96)	1.46 (1.04–2.04)	1.99 (0.84–2.73)	1.51 (0.84–2.73)
Hypertension before or during pregnancy	1.08 (0.78–1.49)	1.2 (0.78–1.84)	1.05 (0.8–1.38)	1.14 (0.75–1.74)	1.02 (0.63–1.65)
Hypertension during pregnancy only	1.08 (0.77–1.51)	1.27 (0.81–2)	1.09 (0.82–1.44)	1.04 (0.66–1.63)	1.14 (0.7–1.87)

*Adjusted for maternal age category, maternal educational status, use of food stamps, use of public insurance, and marital status, as well as family history of ADHD. Maternal BMI, diabetes, and hypertension models were adjusted for MSDP; MSDP, diabetes, and hypertension models were adjusted for maternal BMI. Table displays relative risks with 95% confidence intervals in parentheses. Reference groups for each exposure are non-smokers, BMI < 25, no diabetes diagnosis, and no hypertensive disorders of pregnancy. BMI, body mass index; MSDP, maternal smoking during pregnancy; PP, pre-pregnancy.

### Interaction between body mass index and diabetes

Table 4 evaluates the evidence for additive or multiplicative interactions between the effects of elevated maternal BMI and maternal pregestational and/or gestational diabetes on the risk of ADHD outcomes. Although the results were not statistically significant, the magnitude and direction of the effects provide evidence of some interaction, particularly given that lack of statistical significance may be due to insufficient sample size. The relative excess risk due to interaction (RERI) was 1.21 (95% CI: −0.75, 3.17), meaning that there were some indications that the estimated joint effect on the additive scale of elevated maternal BMI and maternal diabetic status together was greater than the sum of the estimated effects of BMI alone and diabetes alone. The proportion of disease among those with both exposures that is attributable to their interaction (AP) is 0.43 (95% CI: −0.09, 0.96). The corresponding synergy index (SI: ratio between the combined effect and sum of the individual effects) is 3.1 (95% CI: 0.32, 29.6). The measure of interaction on a multiplicative scale, the ratio of relative risks, was 1.73 (95% CI: 0.52, 5.72), meaning that there were some indications that the estimated joint effect on the relative risk scale of elevated BMI and diabetes together was greater than the product of the estimated effects of elevated maternal BMI alone and diabetes alone.

**TABLE 4:** Assessment of interaction between elevated maternal pre-pregnancy BMI (≥25) and maternal diabetes.

	Elevated BMI absent	Elevated BMI present	Effect of elevated BMI within the strata of diabetes
	RR (95% CI)	RR (95% CI)	RR (95% CI)
Diabetes absent	1 (Reference)	1.51 (1.07, 2.13)	1.51 (1.07, 2.13)
Diabetes present	1.07 (0.38, 2.97)	2.79 (1.49, 5.23)	2.61 (0.83, 8.2)
Effect of diabetes within the strata of elevated BMI	1.07 (0.38, 2.97)	1.84 (0.99, 3.45)	
Interaction on multiplicative scale	1.73 (0.52, 5.72)		
RERI	1.21 (−0.75, 3.17)		
AP	0.43 (−0.09, 0.96)		
SI	3.1 (0.32, 29.64)		

AP, proportion attributable to interaction; RERI, relative excess risk due to interaction; SI, synergy index.

### Evaluation of mediation by alterations in inflammatory or neurotrophic proteins

The model predicting ADHD outcomes with perinatal inflammatory protein level as the exposure demonstrated some evidence that high levels of inflammatory proteins, but not moderate levels, were associated with ADHD outcomes (Table 5). While only the model for the “less severe” phenotype (including those on ADHD medication who did not meet ADHD cutoff criteria) was statistically significant, the other ADHD outcomes had a non-significant relative risk of similar magnitude (RR 1.39–1.58). There was also some evidence for association of moderate levels, but not high levels, of neurotrophic protein levels with an increased risk of ADHD outcomes (Table 5). Table 6 demonstrates that maternal BMI and maternal diabetes were not significantly associated with high levels of inflammatory proteins or moderate levels of neurotrophic proteins. Therefore, the final mediation analysis step of simultaneously evaluating the effects of maternal BMI/diabetes and protein levels on ADHD outcomes is unnecessary. The data do not support the hypothesis that effects of these risk factors on ADHD outcomes are mediated through perinatal inflammation.

**TABLE 5:** Adjusted analyses for ADHD outcomes by inflammatory or neurotrophic protein status.

	ADHD 10 or 15	ADHD 10 or 15 AND medication	ADHD 10 or 15 OR medication	ADHD 10	ADHD 15
Inflammation – Moderate	0.98 (0.73–1.33)	1.16 (0.77–1.73)	0.95 (0.73–1.23)	1 (0.69–1.44)	0.93 (0.58–1.5)
Inflammation – High	1.24 (0.86–1.78)	1.43 (0.86–2.38)	1.43 (1.09–1.89)	1.21 (0.76–1.91)	1.37 (0.8–2.34)
Neurotrophins – Moderate	1.35 (1–1.82)	1.53 (1.01–2.32)	1.23 (0.95–1.59)	1.49 (1.03–2.16)	1.34 (0.84–2.15)
Neurotrophins – High	1.05 (0.75–1.47)	1.06 (0.65–1.73)	1.16 (0.88–1.52)	1.21 (0.8–1.81)	0.87 (0.5–1.53)

Table displays relative risks with 95% confidence intervals in parentheses. Reference groups for each exposure are low levels of the protein groups, with low, moderate, and high levels defined in Section “Materials and methods”. Adjusted for maternal age category, maternal educational status, use of food stamps, use of public insurance, and marital status, as well as family history of ADHD.

**TABLE 6:** Adjusted analyses for inflammatory and neurotrophic protein outcome by maternal BMI or diabetes status.

	High inflammatory proteins	Moderate neurotrophic proteins
Maternal PP BMI 25–30	1.01 (0.57–1.80)	1.00 (0.68–1.45)
Maternal PP BMI > 30	1.27 (0.77–2.11)	1.14 (0.82–1.59)
Maternal diabetes	1.41 (0.72–2.76)	0.71 (0.36–1.40)

Table displays relative risks with 95% confidence intervals in parentheses. Reference groups for each exposure are BMI < 25 and no diabetes diagnosis. Adjusted for maternal age category, maternal educational status, use of food stamps, use of public insurance, and marital status, as well as maternal hypertension and maternal smoking during pregnancy.

## Discussion

In this longitudinal follow-up study of an extremely preterm cohort, we demonstrated a relationship between prenatal maternal modifiable risk factors and ADHD at age 10 and 15. After adjusting for socioeconomic variables and family history of ADHD, MSDP and maternal HDP were not related to ADHD outcomes, but elevated maternal BMI and pregestational or gestational diabetes were associated with an approximately 55–65% increase in risk individually. Furthermore, there was evidence of an additive and multiplicative interaction between elevated BMI and diabetes, such that those mothers with both conditions had a 2.8-fold increase in risk of their children developing ADHD compared to those mothers with neither condition. We hypothesized that a common characteristic of these risk factors may be the creation of an inflammatory state prenatally and that maternal inflammation may lead to a neonatal inflammatory state that could mediate neurodevelopmental changes. Contrary to this hypothesis, we did not find evidence that alterations in either inflammatory or neurotrophic proteins in the first week of life mediated long-term ADHD outcomes.

This study is novel in that it specifically evaluates modifiable risk factors for ADHD in an extremely preterm cohort. Prematurity itself has been identified as an independent risk factor for the development of ADHD (Franz et al., 2018), with a two- to three-fold increased risk for those born at <33 weeks gestational age and four-fold increased risk for those born at <26 weeks (Linnet et al., 2006; Johnson and Marlow, 2011). In our recent report on the ELGAN cohort, ADHD was the most frequently occurring psychiatric disorder in individuals born preterm, with a prevalence of 18% at age 15 (Frazier et al., 2022). Perinatal medical and neurological factors contribute to risk of ADHD among individuals born preterm (Indredavik et al., 2010; Leviton et al., 2018), but prior studies in this group have not focused on the contribution of modifiable maternal risk factors.

We capitalized on the longitudinal nature of the ELGAN study to develop a broad ADHD outcome measure to examine ADHD-related clinical symptoms across a spectrum of severities. This outcome captures individuals with symptoms related to hyperactivity/impulsivity or inattention either at age 10 or 15 years. By including both age groups, we are including those individuals who may struggle with symptoms at younger ages but function better during adolescence, as well as those who may be able to manage symptoms more adaptively at younger ages until academic demands outstrip their ability to adapt. We included this broader range of indicators of ADHD outcomes to increase the power of the study to detect associations. We limited our analyses to any ADHD diagnosis, and not individual subtypes (i.e., inattentive, hyperactive-impulsive, or combined) because the small numbers of children in each subgroup substantially limited power and did not allow for robust analyses. The ADHD literature is supportive of this unitary concept of ADHD (Heidbreder, 2015), with factor analyses showing a heavy “common factor” loading (Bernfeld, 2012; Martel et al., 2012).

The potential limitations of our chosen outcome measures are that individuals may be misclassified due to (i) inclusion of individuals with symptoms but not meeting the criteria of clinical impairment, or (ii) by under-inclusiveness by missing individuals whose symptoms are well-managed with appropriate treatment and thus do not meet the cutoffs for the CSI-4 or MINI-KID, both of which require current reporting of symptom counts. To address each of these concerns respectively, we conducted a sensitivity analysis by which we evaluated the relative risks (i) including only those who met the symptom cutoffs for the CSI-4 or MINI-KID and had also been clinically treated for ADHD with medication, and (ii) including anyone who had been treated with ADHD medications even if they did not meet the symptom cutoffs for the outcome measures. The pattern of relative risks was not changed by use of these different outcome groups, and the magnitude of the risk increase was greatest for those most severely affected by ADHD symptoms, that is, those who were treated with ADHD medication and continued to exhibit symptoms consistent with a diagnosis of ADHD.

### Elevated maternal body mass index, maternal diabetes, and their interaction

We found a strong association between elevated maternal pre-pregnancy BMI and risk of ADHD, with similar increase in risk for overweight and obese mothers. Previous studies have also identified this associated risk increase in large population-based cohorts, although typically with evidence of greater risk increase for obese mothers than overweight mothers. For example, a systematic review and meta-analysis including data from 36 cohorts found that risk of ADHD was 30% greater for offspring of overweight mothers and 62% greater for obese mothers (Sanchez et al., 2018). Our study confirms the previously identified increase in odds for parent-identified ADHD criteria on the CSI-4 at age 10 by our group using the same preterm cohort (OR = 1.9 for overweight, OR = 2.3 for obese) (van der Burg et al., 2017). The ELGAN cohort is the only preterm cohort for which the relationship between elevated maternal BMI and ADHD risk has been examined. The current study extends those results by including data from age 15, better adjusting for confounding, especially that due to family history of ADHD, evaluating effect measure modification, and evaluating neonatal systemic inflammation as a potential mediator.

We also found a significant increase in risk for ADHD in children born to mothers with pregestational or gestational diabetes. This has also been reported previously in population-based cohorts, but ours is the first study to demonstrate this association in a preterm cohort. A previous retrospective cohort study, including 333,132 singletons in the United States, identified a 57% increase in risk of ADHD associated with type 1 diabetes and a 43% increase in risk associated with type 2 diabetes (Xiang et al., 2018). In that study, gestational diabetes overall was not associated with increased risk, although an exploratory analysis of gestational diabetes requiring medication treatment yielded an association with a 26% increase in risk. A systematic review and meta-analysis of 15 previous cohort or case control studies confirmed the lack of an association between gestational diabetes and risk of ADHD (OR = 1.01) (Rowland and Wilson, 2021). Two other European population registry studies identified a 35%–60% increase in risk associated with type 1 diabetes (Instanes et al., 2017; Ji et al., 2018).

Ours is the first study in preterm infants to investigate the interaction between elevated maternal pre-pregnancy BMI and maternal diabetes on the increase in ADHD risk in offspring, and we found evidence for both a multiplicative as well as an additive effect of interaction, with the combination of the two increasing the risk 2.8-fold compared to the absence of either. While multiplicative interaction is often reported from a statistical model, it is generally accepted in epidemiologic studies that the additive interaction is most suggestive of a significant biologic interaction between two exposures (Knol et al., 2007). Two previous population-based studies investigated combined effects of maternal pre-pregnancy obesity and maternal diabetes on ADHD risk. A nationwide registry study of all live births in Finland identified increasing risk with increasing maternal BMI to a maximum of 88% increased risk for severely obese mothers, and a 46% increase in risk with pregestational diabetes in normal weight mothers (Kong et al., 2018). When evaluating added risk of pregestational diabetes in different maternal BMI classifications, only the severely obese had a synergistic effect, with a six-fold increase in risk with a combination of severe obesity and pregestational diabetes. However, this study did not report any formal measures of multiplicative or additive interaction. A smaller cohort study of 2,734 children did not identify significant increased risk of ADHD associated with maternal obesity, maternal diabetes, or the combination, although the hazard ratios suggested a possible effect without the statistical power to reach significance (Li et al., 2016).

Several mechanisms have been postulated for how maternal elevated BMI and maternal diabetes may affect fetal neurodevelopment, and the overlap between suggested mechanisms may explain possible synergistic effects between these exposures. Both obesity (Bilbo and Tsang, 2010; van der Burg et al., 2013; Bolton and Bilbo, 2014; Rivera et al., 2015) and diabetes (Chandna et al., 2015) are characterized by an inflammatory milieu that may affect brain development and later neurodevelopmental outcomes. Preliminary evidence does suggest a link between either elevated prenatal inflammatory cytokine levels (von Ehrenstein et al., 2012; Jones et al., 2017) or elevated postnatal inflammatory cytokine levels (Kuban et al., 2017, 2019) and early childhood neurodevelopment. Obesity and diabetes are both associated with metabolic changes such as increasing circulating levels of leptin and insulin, which may adversely impact development (Hendler et al., 2005; Khanh et al., 2014; Rivera et al., 2015). Both conditions are also related to epigenetic changes during fetal development, which may have far-reaching consequences for later outcomes (Archer et al., 2011; Ding et al., 2015; Neri and Edlow, 2015; Ornoy et al., 2015; Sharp et al., 2015). For obese mothers, there also may be effects of lipotoxicity on the developing fetus (Jarvie et al., 2010). Additionally, maternal hyperglycemia is associated with fetal hypoxia and increased oxidative stress (Eidelman and Samueloff, 2002; Biri et al., 2006; Ornoy et al., 2015), both of which may have adverse impacts on fetal neurodevelopment (Wells et al., 2009; Getahun et al., 2013). Mothers with type 1 diabetes, in particular, may have impaired DNA integrity in germ cells (Wang et al., 2010), predisposing the pregnancy and fetal development to adverse outcomes. Maternal diabetes is also associated with other adverse outcomes of pregnancy, which may mediate the long-term effects of exposure, such as pre-eclampsia, infants born large for gestational age, and Caesarian delivery (Wendland et al., 2012; Billionnet et al., 2017).

### Maternal smoking during pregnancy

We did not find an association of MSDP with ADHD outcomes, which is in contrast to much of the literature in population-based studies of epidemiological risk for ADHD (Dong et al., 2018). Neurobiologically, animal studies have demonstrated an association between *in utero* nicotine exposure and increased motor activity in offspring (Ajarem and Ahmad, 1998) as well as impairments in attention, memory, and learning (Levin et al., 1993), possibly due to alterations in hippocampal cholinergic receptors (Yanai et al., 1992). In humans, prenatal smoking exposure has been associated with structural brain changes in the frontal lobe and cerebellum (Ekblad et al., 2010), and neurodevelopmental changes may be mediated through fetal hypoxia (Verhagen et al., 2011) or epigenetic modifications (Toledo-Rodriguez et al., 2010). However, the causal role of MSDP on ADHD outcomes has been questioned, with evidence that associations may be confounded by unmeasured familial factors (D’Onofrio et al., 2008; Agrawal et al., 2010; Skoglund et al., 2014). The only previous study evaluating prenatal risk factors for ADHD within a preterm cohort was a previous report from our group on antecedents of screening positive for ADHD at age 10, which showed a 2.2-fold increase in odds for parent-reported ADHD associated with MSDP (Leviton et al., 2018). However, the previous report used a time-oriented risk model to evaluate approximately 100 variables by univariate analysis to build a multinomial logistic regression model with significant risk factors without an *a priori* theoretical basis for inclusion or exclusion of potential confounders. In particular, the study did not account for potential confounding by familial history of ADHD. In contrast, our study used a theoretical directed acyclic graph to include the minimum sufficient set of confounding variables to include in the adjustment models, including family history of ADHD.

The lack of association between MSDP and ADHD outcomes may indicate that the association is less pronounced or absent in preterm cohorts compared to term cohorts. Alternatively, our cohort may have been better matched between maternal smokers and non-smokers on unmeasured familial factors that might have confounded associations in previous studies (D’Onofrio et al., 2008; Agrawal et al., 2010; Skoglund et al., 2014). A limitation of our study that is true of most previously reported MSDP outcome studies is that smoking history during pregnancy is obtained by maternal report, which is known to be susceptible to underreporting (Dietz et al., 2011). However, it is unlikely that the magnitude of misclassification of smoking status by underreporting would be sufficient to explain the adjusted relative risk of 0.88 seen in our study if there were truly an association with increased risk.

### Maternal hypertensive disorders of pregnancy

We also did not find an association between HDP and ADHD outcomes, in contrast to multiple population-based studies (Maher et al., 2018). Large national patient registry studies in Denmark/Sweden (Wang et al., 2021) and Taiwan (Chen et al., 2021), as well as a recent systematic review and meta-analysis of cohort and case-control studies (Maher et al., 2018), have consistently reported that HDP is associated with an approximately 20%–30% increased risk of ADHD in offspring. Some studies have implicated pregnancy-induced hypertension or preeclampsia (Gumusoglu et al., 2020; Barron et al., 2021; Wang et al., 2021), specifically, while others have demonstrated risk for chronic hypertensive disorders as well (Chen et al., 2021). Multiple potential mediators of the increased risk have been proposed, including exaggerated maternal inflammatory response (Mihu et al., 2015), placental insufficiency (Rätsep et al., 2016; Lawrence et al., 2019), and increased oxidative stress (Hsieh et al., 2012; Ahmad et al., 2019). The lack of association seen in our study may mean that the increased risk is not present in a preterm cohort, where other factors associated with preterm delivery may be more significant contributors to the overall increased prevalence of ADHD in this cohort. Premature birth may be paradoxically protecting the fetus from prolonged exposure to the maternal hypertensive environment. However, the lack of perceived association could be due to the lack of power to detect the effect along with the masking of the effect by a misclassification of individuals in terms of ADHD diagnosis due to the broad outcome measure used. In support of this effect masking, the use of a more stringent outcome measure (the requirement to be treated with ADHD medication) led to an increase in the magnitude of the relative risk into the range of 20%–30% increased risk (Table 3) seen in previous studies, although the 95% confidence interval of the estimate continued to extend below one.

### Perinatal inflammation

Because of the postulated role of inflammatory pathways in mediation of long-term effects of these *in utero* exposures, we evaluated the connection between both inflammatory cytokines and proteins as well as neurotrophic proteins, with ADHD outcomes. We have previously shown associations between these postnatal protein levels and neurologic and cognitive outcomes (Kuban et al., 2017, 2019). We found that there was a moderate association between high levels of inflammatory proteins or moderate levels of neurotrophic proteins and ADHD at age 10 or 15, but no association between these proteins and maternal BMI or diabetes. Therefore, at the level of neonatal inflammatory markers, elevated risk of ADHD in the preterm-born child to mothers with elevated BMI or diabetes does not show evidence of mediation through inflammatory pathways. However, the association of neonatal inflammatory proteins and neurotrophic proteins with ADHD outcomes is a novel finding, and further work to evaluate methods of modifying these neonatal outcomes or moderating their effect on ADHD is warranted. Also, the demonstrated relationship with neurotrophic proteins is complex, with moderate but not high levels of neurotrophins associated with increased risk. It may have been expected that neurotrophic proteins would confer protection against neurodevelopmental outcomes. However, there is likely a complex interrelationship between inflammatory and neurotrophic proteins with an increase in neurotrophins acting as a compensatory response, perhaps insufficient to decrease the risk of ADHD conferred by perinatal inflammation. Importantly, we did not have data on maternal circulating inflammatory markers during pregnancy or within the intrauterine environment, so it remains possible that exposure to these cytokines may play a role in the link between prenatal exposures and ADHD outcomes.

### Strengths and limitations

The strengths of this study include its prospective design, the large number of children, the enrollment based on gestational age, the long-term longitudinal follow-up, ascertainment of ADHD by study personnel who were unaware of participants’ prenatal exposures, and the innovation of evaluating multiple modifiable risk factors specifically in the ELGAN population. The adjustment for socioeconomic factors including maternal age, maternal educational status, use of food stamps, use of public insurance, and marital status, as well as family history of ADHD, minimizes the confounding by these pre-existing risk factors for both the investigated exposures as well as ADHD outcomes.

The limitations of our study include the use of maternal self-report for pre-pregnancy weight and smoking status during pregnancy. Our reliance on maternal report may have led to misclassification; to the extent that misclassification was non-differential with regard to exposures, it would have led to under-estimation of the strength of associations, possibly masking associations with MSDP and HDP. Our outcome measure has the benefit of incorporating data from two different time points in development, potentially increasing the power of our study to detect a relationship. However, the limitation of the CSI-4 as a less validated measure of ADHD diagnosis is acknowledged (Collett et al., 2003). This is mitigated somewhat by the inclusion of a validated diagnostic instrument at age 15 and the strengthening of exposure-outcome relationships when the outcome was limited to those that are being treated with ADHD medications. While ADHD outcomes were relatively common, the incidence was low enough that analysis by ADHD subtype was not feasible due to unstable risk estimates from our statistical models. Another limitation of our study was the relatively high attrition rate at age 15 years. One of the main challenges with longitudinal studies is retention of participants (Doyle and Anderson, 2018), and our retention rate of 58% at age 15 is consistent with other studies that have followed preterm cohorts into their teenage years (Johnson et al., 2019). This loss was mitigated somewhat by including data from the larger sample at age 10.

Our analysis of the effect of maternal diabetes is limited by the lack of ability to distinguish between type 1 and type 2 diabetes in the individuals with pre-pregnancy diabetes. The majority of our diabetes sample (36/56; 64%) had gestational diabetes, and epidemiologic reports on pregnancy in the United States during the time period of this cohort (Albrecht et al., 2010) identified a slight preponderance of type 2 diabetes compared to type 1 (8% vs. 6%) in mothers with pre-pregnancy diabetes. Therefore, we would estimate that only approximately 15% of our diabetes sample would have type 1 diabetes, which may be considered a less modifiable risk factor. Also, although the presence of diabetes is perhaps more “modifiable” in the case of type 2 diabetes, the management and control of diabetes during pregnancy is a potentially modifiable target in all cases. Therefore, it is justifiable to include all maternal diabetes in this analysis.

Finally, there is no data available on the levels of inflammatory and neurotrophic proteins in the general population, so the cutoff for a clinically relevant elevation in these proteins is unknown. We have previously reported extensively on relationships with perinatal inflammation in this extremely low gestational age cohort using the dichotomization at the 75th percentile, indicating that this is a clinically significant cutoff in this population (e.g., Kuban et al., 2017, 2019; Belfort et al., 2022; Eaves et al., 2022). However, it is possible that using a different cutoff would identify different relationships to the ADHD outcomes.

Because this is a study of preterm births, the effect of maternal factors on ADHD may be underestimated given that the inherent study design is controlling for gestational age by restriction of the population studied. It is quite possible that preterm birth itself, or other factors associated with preterm birth, may mediate a relationship between the risk factors and ADHD outcome. Any indirect effects of the risk factors on ADHD, mediated by preterm birth, would not be evident in these analyses. This may partially explain the lack of an effect of MSDP or HDP on ADHD in this population.

## Conclusion and future directions

While MSDP and maternal HDP were not associated with significant risk increase for ADHD in a large preterm cohort at age 10 or age 15, maternal pre-pregnancy BMI and maternal diabetes were both associated with elevated risk, independent of other socioeconomic variables. Also, we found moderate evidence of an association between high levels of perinatal inflammation or moderate levels of neurotrophic proteins and ADHD, but these did not mediate the elevated risk associated with maternal BMI or diabetes. Importantly, maternal overweight/obesity status and diabetes interacted to elevate risk for ADHD in offspring above what would be expected based on individual exposures. Future efforts will be needed to determine if obesity prevention, diabetes management strategies or interventions that modify perinatal inflammation, either during conception planning or early in pregnancy, can mitigate the risk of long-term neurodevelopmental outcomes such as ADHD. Also, further work is needed on investigating potential mediators by which these exposures may increase risk, including epigenetic effects, oxidative stress, metabolic effects, and fetal hypoxia.

## Data availability statement

The raw data supporting the conclusions of this article will be made available by the authors, without undue reservation.

## Ethics statement

The studies involving human participants were reviewed and approved by the institutional review boards of all participating institutions. Written informed consent to participate in this study was provided by the participants’ legal guardian/next of kin.

## Author contributions

DC, EJ, JF, IJ, SK, KR, RJ, SH, HS, KK, RF, and TO’S contributed to the conceptualization, investigation, and writing – review and editing. JF, HS, RF, and TO’S contributed to the data curation. DC, KR, EJ, and TO’S contributed to the formal analysis. JF, RJ, SH, HS, KK, RF, and TO’S contributed to the funding acquisition. DC, EJ, KR, and TO’S contributed to the methodology. DC, JF, RJ, SH, HS, KK, RF, and TO’S contributed to the project administration. DC, KR, and TO’S contributed to the writing – original draft. All authors contributed to the article and approved the submitted version.
