# Supplementary material for: Association of prenatal modifiable risk factors with attention-deficit hyperactivity disorder outcomes at age 10 and 15 in an extremely low gestational age cohort
Source: Front Hum Neurosci. 2022 Oct 20;16:911098. doi: 10.3389/fnhum.2022.911098 (PMC9630552; doi:10.3389/fnhum.2022.911098)
Supplement: Supplementary file 1 [file Data_Sheet_1.PDF]

Supplementary Material

Table S1. Sensitivity analysis repeating Poisson regression models as in Table 3, but including all participants regardless of IQ and adding full-scale IQ as covariate to adjust for confounding by IQ.

	ADHD 10 or 15	ADHD 10 or 15 AND Medication	ADHD 10 or 15 OR Medication	ADHD 10	ADHD 15
MSDP	1.01 (0.72-1.43)	0.79 (0.48-1.32)	1.1 (0.83-1.47)	1.16 (0.77-1.75)	1.25 (0.77-2.01)
Maternal PP BMI 25-30	1.71 (1.29-2.28)	1.6 (1.07-2.4)	1.47 (1.14-1.89)	2 (1.4-2.86)	1.93 (1.27-2.94)
Maternal PP BMI > 30	1.44 (1.07-1.94)	1.67 (1.14-2.47)	1.57 (1.24-1.99)	1.82 (1.28-2.6)	1.79 (1.16-2.75)
Maternal Diabetes	1.6 (1.12-2.29)	1.93 (1.2-3.12)	1.49 (1.1-2.01)	1.78 (1.13-2.83)	1.67 (0.99-2.85)
Hypertension Before or During Pregnancy	1.06 (0.79-1.44)	1.28 (0.86-1.89)	1.05 (0.81-1.34)	1.06 (0.72-1.55)	1.1 (0.71-1.68)
Hypertension During Pregnancy Only	1.09 (0.8-1.49)	1.38 (0.92-2.08)	1.07 (0.83-1.39)	1.03 (0.68-1.56)	1.28 (0.83-1.97)

BMI = Body Mass Index; MSDP = Maternal Smoking During Pregnancy; PP = Pre-pregnancy
Adjusted for IQ, maternal age category, maternal educational status, use of food stamps, use of public insurance, and marital status, as well as family history of ADHD. Maternal BMI, diabetes, and hypertension models were adjusted for MSDP; MSDP, diabetes, and hypertension models were adjusted for maternal BMI.
